# Use of Cephalic Vein for Venous Anastomosis in Head and Neck Reconstruction: A Case Series

**Published:** 2020-07-06

**Authors:** Mohammad Fazlur Rahman, Muhammad Asif Ahsan, Safdar Ali Shaikh, Muhammad Ubaid Khan, Sami ullah

**Affiliations:** ^a^Department of Plastic Surgery, Aga Khan University Hospital, Karachi, Pakistan; ^b^Department of Plastic Surgery, Cancer Foundation Hospital, Karachi, Pakistan; ^c^Shaukat Khanum Memorial Cancer Hospital and Research Centre, Peshawar, Khyber Pakhtunkhwa, Pakistan

**Keywords:** head and neck, free flaps, cephalic vein, recipient, venous anastomosis

## Abstract

**Purpose:** To describe the use of the cephalic vein as a recipient vessel for venous outflow in head and neck reconstruction. The cephalic vein is used as a vessel in cases where there is a paucity of veins in the neck. This may be due to previous surgery, previous radiation therapy, or advanced cancer. The cephalic vein may also be used to salvage a failing free flap. **Methods:** It is a retrospective review of 230 free flaps that had been used in head and neck reconstruction performed from July 2014 to July 2018 by a single surgeon. **Results:** There were 6 patients in whom the cephalic vein was used as a recipient vessel. The cephalic vein was used in 2 cases where a tumor was involved with the internal jugular vein, in 1 case where a previous neck dissection had been done, in 1 case where internal jugular vein had iatrogenic damage, and in 2 cases a salvage procedure was done. In all cases, the cephalic vein was rotated above the clavicle. The cephalic vein easily reached the free flap vein and had a good caliber. There were no failures in any of the patients. **Conclusion:** The cephalic vein is a good option for venous anastomoses in patients in whom there is a paucity of recipient vessels in the neck.

Microvascular free flaps have become increasingly essential for patients requiring head and neck reconstruction, with success rates greater than 95%.^1^ As success rates have increased, surgeons have started operating on more complex cases. Complexities include previous surgeries, previous radiotherapy, more advanced cancer, or a combination of these modalities.^2^ Radiotherapy leads to intimal fibrosis and arteriosclerosis of the vessels. Previous surgery and extensive disease may lead to compromised vessel. Reconstructing free flaps in such cases is always a challenge as there is vessel depletion/unsuitability, particularly of veins. Commonly used veins that include ipsilateral external jugular vein (EJV) and internal jugular vein (IJV) are usually unavailable, thrombosed, or unsuitable for anastomosis.^3^ In such cases, the surgeon is forced to look for other options. These include contralateral neck veins, use of a vein graft, or an alternate recipient vein.^4^

A good option for alternate recipient vein is the cephalic vein. Use of the cephalic vein in microsurgery was first described by Hallock.^5^ Horng and Chen^6^ called it the lifeboat for head and neck reconstruction. The first series was published by Kim and Chandrasekhar.^7^ Here, we report our experience with using the cephalic vein as a recipient vein in cases of head and neck cancer reconstruction.

## ANATOMY AND SURGICAL TECHNIQUE

The cephalic vein is present in the forearm fascia and ascends along the radial border of the radius from the forearm.^8^ In the arm, it ascends along the lateral bicipital groove on the lateral side of biceps muscle. It then enters the deltopectoral groove to lie between the pectoralis major and deltoid muscle.^9^ In the upper part, the thracoacromial artery and its branches lie in close proximity to the cephalic vein. It pierces the costocoracoid fascia and drains into the axillary vein. The place where the cephalic vein enters the costocoracoid fascia is the pivot point of the flap.^10^

An open technique is used to harvest the cephalic vein. This is done to prevent any postoperative compression on the vessel. The groove between the pectoralis major and the deltoid muscle is palpated below the acromion. Incision is made over the deltopectoral groove. Once deep fascia is opened, the vein can be visualized between the pectoralis major and deltoid muscle. Branches of the thoracoacromial artery may be present here and need to be saved. Retrograde dissection is then done, and the vein is followed into the upper arm and mobilized. Measurements are made from the free flap vessel to the point of rotation of the vein. The vein is dissected distally for 2 to 3 cm more than the required length from the point of rotation. Once enough length of the vein is obtained, the proximal end of the vein in the arm near the elbow is ligated. The vein is rotated upward in the semicircular fashion above the clavicle. This is done to prevent kinking of the vein.

## MATERIALS AND METHODS

Data were collected retrospectively from the medical record of the patients. Patients’ confidentiality was maintained by not including the name and address of the patients in the performa, and the patients were addressed by their serial numbers. There were a total of 230 free flaps done from July 2014 to July 2018 by a single surgeon. Of these, there were 6 cases that required use of the cephalic vein (Table 1). All the patients requiring the cephalic vein were male. Their age range was from 30 to 56 years (mean = 46 years). All patients had squamous cell carcinoma. Four patients required soft-tissue reconstructions and 2 required bony reconstruction with soft tissue. Of the soft-tissue reconstructions, 3 patients had reconstruction with anterolateral thigh flap and 1 patient had reconstruction with lateral arm free flap. Both bony reconstructions were done by free fibular flap. Arterial anastomosis was done with lingual artery in 4 cases and with superior thyroid artery in 2 cases. The cephalic vein was used for anastomoses primarily in 3 cases as the sole vessel, as there were no other vessels suitable. In one case, it was used for a second anastomosis in the primary surgery to enhance venous outflow as the flap was large. In 2 cases, the cephalic vein was used to salvage flaps after venous congestion. In the first case, initial anastomosis was end to side into the IJV. Thrombosis occurred 2 hours after surgery, and revision was done using the cephalic vein. In the second case, 2 anastomoses were done, one to the EJV and second to the IJV. After 18 hours, venous congestion occurred. Anastomosis was revised to the EJV and as an adjunct the second vein was anastomosed to the cephalic vein to ensure survival of the flap.

## CASE 1

**Figure 1:**
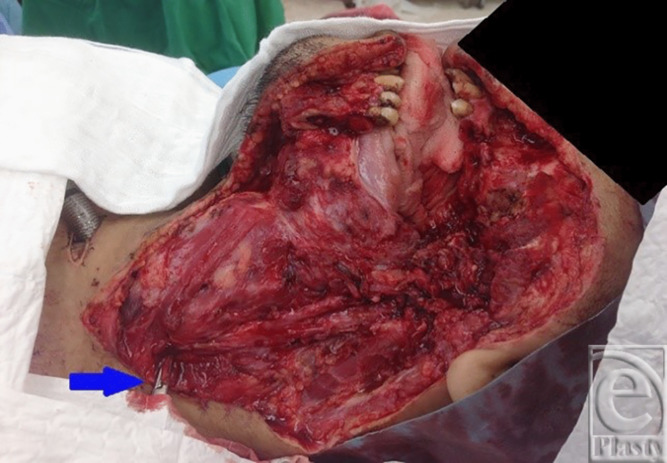
Ligated internal jugular vein.

**Figure 2:**
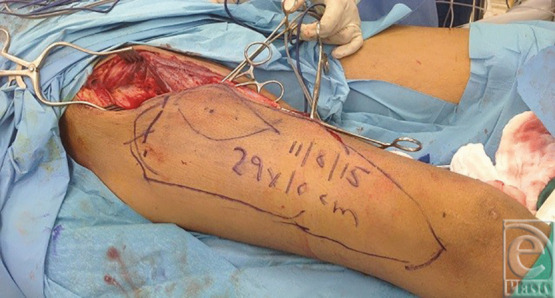
Harvesting of the anterolateral thigh flap.

**Figure 3:**
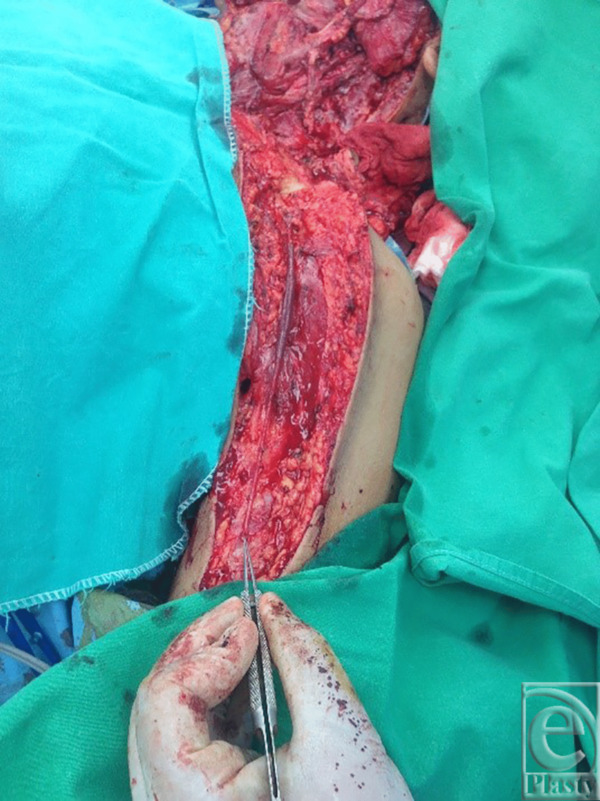
Cephalic vein harvested.

**Figure 4:**
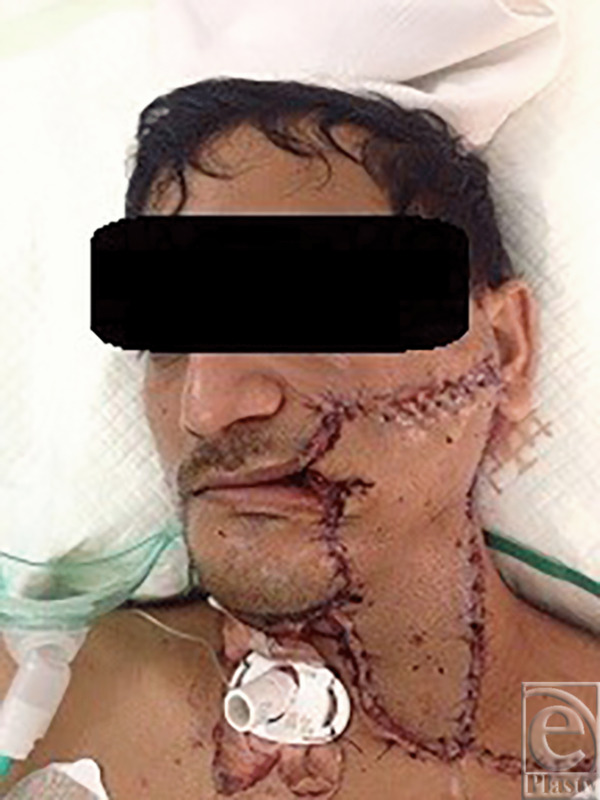
Postoperative day 4.

A 38-year-old man underwent excision of squamous cell carcinoma of the cheek and neck and reconstruction with a free right anterolateral thigh flap. The tumor was large and the IJV was ligated at the base of neck (Fig. 1). A large 29 × 10 cm anterolateral flap was harvested (Fig 2). The artery was anastomosed to the lingual artery. For venous anastomosis, the cephalic vein was harvested and anastomosis done to the flap vein (Fig 3). The patient did well postoperatively (Fig 4).

## CASE 2

**Figure 5:**
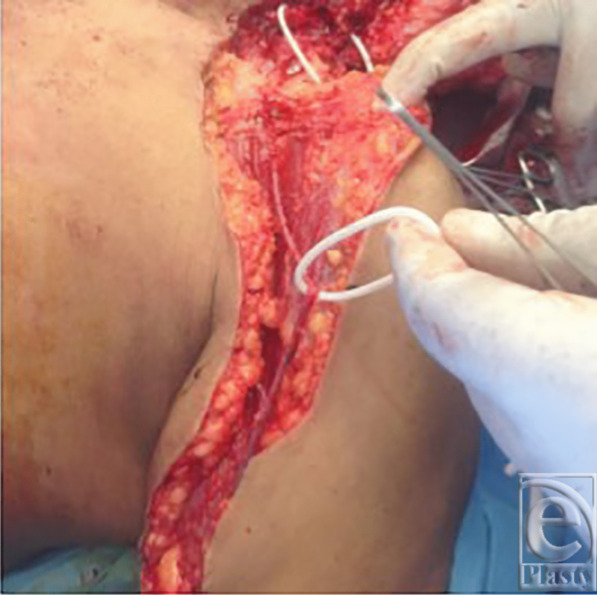
Cephalic vein.

**Figure 6:**
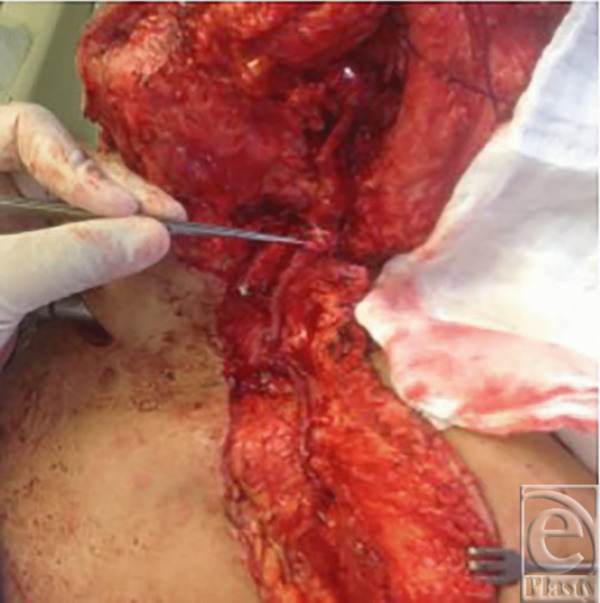
Cephalic vein rotated for anastomoses.

A 50-year-old man underwent resection of squamous cell carcinoma of the mandible. A free fibular flap was done with arterial anastomosis to the lingual artery and 2 venous anastomoses (one end to end to the EJV and second end to side to the IJV). After 18 hours, the flap started getting congested. On reexplanation, the vein anastomosed to the IJV was thrombosed while the vein anastomosed to the EJV was partially thrombosed. Venous anastomosis to the EJV was revised. To ensure flap survival, it was decided that a second anastomosis should be done. The cephalic vein was then harvested and end-to-end anastomosis was done between the cephalic vein and the second vein (Figs 5 and 6). The flap survived and recovery was uneventful.

## DISCUSSION

The IJV, its branches, and the EJV are the most commonly used recipient vessels in head and neck free flaps. These veins have good caliber and are reliable.^11^ In patients with advanced diseases, recurrences, or needing salvage procedure, these vessels may be compromised and an alternate vessel may be required for venous outflow. The cephalic vein is a good alternative for such cases. The cephalic vein has many advantages, which have been previously published.^7^^,^^10^^,^^12^ The vessel has a long pedicle that can easily reach the neck. It is easily dissectible and has a large diameter suitable for microvascular anastomosis. It also requires only one anastomosis. It is also present outside the zone of previous surgery and radiation therapy. All these make the cephalic vein suitable for anastomosis. Different techniques have been used to harvest the cephalic vein including the modified “stepwise” technique.^13^ We prefer the open technique, as the vessel is easy to handle and any chance of injury to the vein while harvesting it is minimum. To prevent vessel kinking, we turn the vessel in a semicircular fashion. Compression of the vein over the clavicle has been noted.^12^ To prevent compression postoperatively, a small groove is made on the clavicle to place the vessel and skin closed in a single layer. Hanasono et al^14^ reported loss of the EJV and IJV in 16% of patients after radiation therapy or surgery. Herle et al^15^ reported a higher risk of free flap failure after radiation therapy. In our study, there was no case in which the cephalic vein was used in free flaps after radiation therapy. The reason being in such cases, we always prefer the contralateral side of the neck. We did use the cephalic vein in one case where neck dissection had been done previously, but patient had not undergone radiotherapy. We used the cephalic vein for anastomosis in 2 cases where the tumor was extensive. In one of the cases, the tumor was abutting the vessel and the venous flow was decreased. In the other case, the IJV was clamped at the base of the neck just above the clavicle. Using a vein graft here would have required 2 anastomoses, so we preferred to use the cephalic vein. In 2 cases, the cephalic vein was used as a salvage procedure after venous congestion. In one of the cases, only the cephalic vein was used for venous outflow but in the second case the cephalic vein was used as a second vein to ensure flap survival. Drawbacks of using the cephalic vein include additional surgical incision,^16^ additional scar, and thin wall of the cephalic vein. Reid and Taylor^17^ found the absence of the vein in 2 of 50 cases, and Jacobson et al^12^ reported small size of the vein in one of 10 cases.

## CONCLUSION

The cephalic vein is a viable option as a recipient vein in free flaps being used in vessel-depleted necks during head and neck reconstruction. It should certainly be considered an option as opposed to the contralateral IJV in cases where there is a high likelihood of recurrence of the carcinoma, so as not to complicate future prognosis and treatment.

## Figures and Tables

**Table 1:** Demographics and vascular anastomosis*

	Sex	Age	Defect area	Flap	Arterial anastomosis	Venous anastomosis	Reason for choosing the cephalic vein
1	Male	38	Cheek, lips, and neck	ALTF 29 × 10 cm, 2 perforators	Lingual artery	Cephalic vein	IJV ligated above the clavicle because of tumor involvement
2	Male	50	Mandible defect	Fibula with lining 6 × 4 cm	Superior thyroid artery	Two veins done, one to the EJV and second to the IJV. Anastomosis revision, one vein to the EJV and second to the cephalic vein	After 18 h, the flap became congested. The patient was taken back to the OR; vein to the IJV thrombosed and to the EJV partially thrombosed
3	Male	30	Cheek lining and cover	ALTF 14 × 7 cm	Lingual artery	Cephalic vein	The IJV had decreased flow due to tumor involvement
4	Male	55	Palate and inner lining	ALTF 15 × 6 cm	Lingual artery	Cephalic vein	Previous neck dissection 10 y back. No suitable veins in the neck
5	Male	48	Mandible defect with both internal lining and external cover required	Fibula lining and cover 20 × 10 cm flap	Superior thyroid artery	Two venous anastomoses: the first vein to the EJV and the second vein to the cephalic vein	Iatrogenic injury to the IJV. Two venous anastomoses done as the flap was large
6	Male	56	Palate, maxilla, and lining	Lateral arm 15 × 6 cm	Lingual artery	Vein done to the IJV. Venous congestion after 2 h, anastomoses revised with the cephalic vein	Iatrogenic injury to the IJV

*ALTF indicates anterolateral thigh flap; IJV, internal jugular vein; EJV, external jugular vein; OR, operating room
